# MAGIC maize: a new resource for plant genetics

**DOI:** 10.1186/s13059-015-0713-2

**Published:** 2015-09-11

**Authors:** James B. Holland

**Affiliations:** USDA-ARS Plant Science Research Unit, Department of Crop Science, North Carolina State University, Raleigh, NC 27695-7620 USA

## Abstract

A multiparent advanced-generation intercross population of maize has been developed to help plant geneticists identify sequence variants affecting important agricultural traits.

## Introduction

Maize is a staple crop worldwide, and its cultivated forms vary dramatically in their environmental adaptation and visible appearance. Underlying the incredible phenotypic diversity is a very high level of genome sequence variation — the average rate of single-nucleotide polymorphism (SNP) variation in maize is ten times greater than it is in humans [1]. In addition to SNP variation, maize varieties exhibit substantial structural differences — namely, copy-number variations and a range of structural variations, which include the presence or absence of expressed genes in different maize genomes [2].

It is likely that any two maize lines from different geographic regions have at least one type of sequence difference in most of their genes. Given this high level of sequence-level variation between maize varieties, how can we know which particular sequence differences contribute to the observed phenotypic differences?

Maize geneticists have attacked this problem by using various techniques, including mutant analysis, linkage mapping and transposon tagging, with notable success in identifying genes with large-effect mutations, such as the components of seed-color pathways. Identifying genes that have smaller effects on quantitatively measured traits is more difficult — many quantitative trait locus (QTL) mapping studies have been conducted in maize, but few have pinpointed causal genes. This is because many of the typical approaches use populations that are too small and have insufficient recombination events.

As the resolution of linkage mapping in modestly sized populations derived from two-parent crosses is usually insufficient to identify precisely the causal variants underlying QTLs, the creation of inbred lines derived from multi-parent cross designs has been used to address these problems. These alternative genetic mapping strategies with higher resolution include association analysis [3], advanced intermated lines [4] and multiple biparental family sets, such as the maize nested association mapping (NAM) panel [5]. These designs require trade-offs among the amount of genetic variation sampled, the resolution of genetic mapping, the confounding effects of population substructure, and the effort required to generate the mapping population. Now a paper by Della’Aqua and colleagues published in *Genome Biology* presents an analysis of the first multiple-parent advanced-generation inter-cross (‘MAGIC maize’ or ‘MM’) population in maize [6]. The resulting population offers some unique properties to facilitate the genetic analysis of complex traits, and this design, combined with the genetics resources available in maize, provides a powerful genetic resource. Other plant MAGIC populations have been developed for analysis of complex traits in *Arabidopsis*, wheat and rice [7–9].

## The advantages of mapping with MAGIC

Diversity panels often have substantial sub-population genetic structure resulting from gathering together geographically distinct lines with varying levels of pedigree relationships [3]. Subgroups within the diversity panel can differ for mean trait values and also for allele frequencies at many loci, leading to false-positive marker-trait associations due to population sub-structure instead of close linkage of markers to causal variations. Statistical methods help to remove the confounding effects of population structure on association tests, but at a cost of reduced power of association testing in some cases. MAGIC populations eliminate this population sub-structure, producing stable, homozygous mapping lines by employing several generations of inter-mating following the initial crosses of the founder lines, and by avoiding selection during self-fertilization. The multiple inter-mating generations have the added useful effect of introducing more recombinations along the chromosomes within the population, meaning that the chromosome blocks inherited by each individual mapping line are reduced in size compared with those of the parent genomes, thus allowing geneticists to better uncouple the effects of linked genes.

The MM population that has been developed currently comprises 529 inbred lines, which were derived from intercrossing eight inbred founder lines to produce a maize population whose genomes represent reshuffled combinations of all eight founders; as genotyping continues, the authors plan to release approximately 1000 more lines.

Although an eight-founder population does not sample as much allelic diversity as a diversity panel, MM ensures that the sampled alleles are sufficiently replicated to allow the statistical estimation of their effects. A diversity panel will capture many more rare alleles, but their rarity makes accurately measuring their effects much more difficult. At the other extreme, NAM uses a common reference parent for all of the crosses, resulting in the reference parent alleles being sampled many more times than those of other founders, which is less statistically efficient (although it provides the substantial advantage of conferring better adaptation on the resulting crosses with unadapted parents). Furthermore, QTLs that contribute to the differences among biparental families can be more difficult to detect in designs such as NAM, whereas the MAGIC design avoids the confounding effect of family structure on QTL inheritance. Finally, a wider range of epistatic interactions can be tested in the MAGIC design because a particular haplotype of a founder in one genomic location occurs in combination with the haplotypes of many other founders at different genomic regions.

## Resolving QTL to genes

Della’Aqua and colleagues developed this MAGIC maize population and directly genotyped all of the progeny lines using a moderate-density SNP array with approximately 50,000 markers. In addition, they sequenced the parental lines to generate approximately an additional 30 million SNPs. Using statistical methods originally developed for similar mouse studies, the authors identified the inherited founder haplotype of each mapping line at each genomic window defined by a set of informative markers. This allowed them to impute very accurately the additional SNPs within those intervals, and to supplement QTL mapping based on founder haplotype inheritance (in which each founder is modeled with a unique QTL effect at each local genome region) with mapping based on identity-in-state at the individual SNPs (which assumes biallelic effects shared between founders if they carry the same SNP at a particular site). The authors performed the SNP association tests within intervals of interest defined by the haplotype-based QTLs, providing a sufficiently high resolution to dissect each QTL and identify those candidate genes most likely to contribute to the observed effect. In principle, these tests could also be conducted genome-wide in follow-up studies.

A major goal of complex-trait genetics is to resolve QTLs to underlying causal genes (or sequence variants, not all of which are coding genes). Besides the SNP association tests within QTL intervals, Della’Aqua and colleagues also used a novel approach of searching for genes whose transcription patterns matched the founder allele QTL effects. QTL mapping estimates the haplotype-to-phenotype relationship, whereas the transcription data are used to estimate the expression level of each gene within each founder haplotype — the gene-within-haplotype-to-transcriptome relationship. By hypothesizing that the *cis*-effects of the local haplotypic region of each founder on the expression of genes within the same region might cause some part of the phenotypic variation, the authors use the correlation between gene expression within each founder haplotype and the haplotype effect on the trait to identify genes following this pattern. This approach can miss genes that affect the trait by means other than by direct expression variation, but it is useful in narrowing down to the more likely causal genes within a QTL interval.

In addition to identifying expression variation related to QTL effects, Della’Aqua and colleagues also demonstrated at least one instance in which structural variation appeared to be related to a QTL effect on grain yield. They identified a QTL in which two founders contributed a low-yield QTL effect; within this interval, a cluster of 24 genes in a 2.5-Mbp region had low expression within those same two founder haplotypes, and finally both of those founders (but no others) entirely lacked sequence reads within this region. This suggests that a large sequence deletion involving numerous genes carried by these two founders results in reduced yield. The authors did not directly confirm the sequence deletion, but, if this result holds, it will support other evidence that large-scale structural variants in maize can affect yield and perhaps that the complementation of such variants in crosses between distinct lines contributes to hybrid vigor [10].

## Abbreviations

MAGIC: Multiparent advanced-generation intercross
NAM: Nested association mapping
QTL: Quantitative trait locus
SNP: Single-nucleotide polymorphism
